# Gender differences in mental health profiles among Chinese adolescents: a multiple-group latent profile analysis

**DOI:** 10.1186/s40359-026-05063-7

**Published:** 2026-06-23

**Authors:** Jichang Guo, Yanpei Pan, Yan Zhao, Xiang Zhang

**Affiliations:** 1 School of Education Science, Minzu Normal University of Xingyi, Xingyi, China; 2 School of Literature and Journalism, Minzu Normal University of Xingyi, Xingyi, China

**Keywords:** Dual-factor model of mental health, Latent profile analysis, Adolescents, Gender differences, Mental health profiles

## Abstract

**Background:**

Adolescent mental health crises are pervasive globally, with notable gender differences, yet existing research using the Dual-Factor Model (DFM) of mental health often relies on oversimplified cutoff scores, limiting insights into heterogeneous profiles. This study aimed to identify latent mental health profiles among Chinese adolescents and examine gender differences using latent profile analysis (LPA) and multiple-group LPA within the DFM framework.

**Methods:**

A total of 1,682 Chinese adolescents (Mage = 14.37, SD = 1.23; 55.29% males) completed the *Center for Epidemiological Studies Depression Scale* (CES-D) and *Satisfaction With Life Scale* (SWLS). LPA was employed to classify latent mental health profiles, and multiple-group LPA was used to compare profile structures, proportions, and symptom severity between genders through nested model comparisons.

**Results:**

This study confirmed three optimal mental health profiles in the total sample: (1) mental health profile (62.5% of participants), characterized by low psychological distress across somatic complaints, depressed affect, and positive affect, alongside high life satisfaction; (2) troubled arousal profile (30.5%), marked by moderate subclinical distress and mild impairment in life satisfaction; and (3) severe distress profile (7.0%), exhibiting severe psychological distress across all domains and profound life dissatisfaction. Gender-specific analyses revealed structural differences: males were classified into three profiles consistent with the total sample, while females were optimally categorized into two profiles. Females were disproportionately represented in distressed profiles and showed significantly higher symptom severity in the severe distress profile, particularly in somatic complaints, depressed affect, and life dissatisfaction.

**Conclusion:**

This study confirms three distinct mental health profiles among Chinese adolescents, with significant gender differences in profile structure, proportions, and symptom severity. Females are more vulnerable to mental health distress, highlighting the urgent need for gender-sensitive mental health interventions. These findings advance the understanding of adolescent mental health in China and provide a foundation for targeted screening, prevention, and intervention strategies. The DFM-based LPA approach provides a complementary person-centered approach to traditional assessments, enabling the identification of at-risk adolescents who may be overlooked by conventional methods.

## Introduction

Contemporary epidemiological studies highlight a pervasive and worsening adolescent mental health crisis. Cross-national data indicate that 20%~25% of young people experience clinically significant psychological distress [1]. Longitudinal studies further reveal alarming trends among females, as emotional disorders have shown marked increases over the past decade [2, 3]. This underscores the urgent need for sustained research attention to adolescent mental health. Suldo and Shaffer [4] proposed mental health encompasses not just the absence of mental illness but also positive constructs (e.g., subjective well-being, social functioning). Building on this, the Dual-Factor Model (DFM) of mental health [5] operationalizes this holistic framework by integrating psychopathology and flourishing emotional/social functioning, emphasizing proactive cultivation of positive functioning to support adolescent development. Grounded in this perspective, early mental health intervention yields long-term benefits for personal and social developmental trajectories.

### Dual-factor model of mental health

Introduced by Greenspoon and Saklofske [6], the DFM represents a paradigm shift by integrating subjective well-being and psychopathology as distinct yet complementary dimensions. It categorizes individuals into four subgroups: (1) *complete mental health* (high well-being, low psychopathology); (2) *vulnerable* (low well-being, low psychopathology); (3) *symptomatic but content* (high well-being, high psychopathology); (4) t*roubled* (low well-being, high psychopathology), based on the combinations of psychopathological and positive psychological indicators.

Within the DFM of mental health, negative psychological indicators are predominantly operationalized as psychopathological symptoms, which are conventionally classified into two core categories: internalizing and externalizing problems [4, 7, 8]. Internalizing problems denote inward-oriented emotional and cognitive impairments, including depression, anxiety, perceived stress and general psychological distress. Externalizing problems, by contrast, entail outward-directed maladaptive behaviors such as aggression, rule-breaking conduct and problematic internet use. Positive psychological indicators, on the other hand, are anchored in the construct of subjective well-being, which is operationalized in empirical research either concisely or comprehensively. The concise operationalization encompasses core dimensions of life satisfaction and positive affect [4, 7], with negative affect occasionally included as a complementary indicator for affective assessment balance. The comprehensive operationalization frames subjective well-being as a multidimensional complete mental health construct that integrates emotional well-being, psychological well-being and social well-being [9–11]. Emotional well-being is defined by positive emotional experiences and subjective happiness, psychological well-being encompasses eudaimonic facets such as self-acceptance, personal growth and a sense of life purpose, and social well-being includes indicators of adaptive social functioning like social integration and social contribution. For the sake of research feasibility and measurement efficiency, many empirical studies have also adopted simplified operationalizations in practice, using depression as the sole proxy for negative psychological indicators and life satisfaction as the exclusive measure of positive psychological indicators [8, 12].

Over two decades, robust empirical support for the DFM has emerged across populations (children, adolescents, adults) [6, 13, 14]. Longitudinal studies validate these subgroups and their unique academic/social functioning outcomes [4, 13], while Chinese sample studies confirm the model’s stability and predictive value for academic self-efficacy and emotional regulation [15, 16]. A systematic review found 85% of DFM studies support the four-group structure, with *complete mental health* as the most prevalent category [8] .

A critical limitation is the DFM’s reliance on cutoff scores to dichotomize continuous psychopathology and well-being measures. Magalhãesnoted arbitrary cutoffs oversimplify mental health complexity, risking misclassification of boundary cases [8]. Petersen et al.emphasized reduced sensitivity to nuanced variations, obscuring longitudinal state transitions [17]. Furthermore, Moore et al. [18] further highlighted inconsistent cutoff criteria across studies, reducing subgroup prevalence comparability. Notably, the DFM’s four-group structure remains robust across adolescent samples [6, 19], but cutoff score controversies persist. This necessitates nuanced methodologies like Latent Profile Analysis (LPA) to enhance precision and clinical utility.

### Latent profile analysis for the DFM

Kim et al. [20] criticized the DFM’s dichotomous cutoff approach for artificially enforcing a four-group solution that fails to capture adolescent mental health complexity. In response, studies have adopted data-driven person-centered methods (e.g., latent class analysis (LCA), LPA), which identify subgroups based on multi-indicator score patterns and allow flexible group numbers [21–23]. These methods now surpass traditional unidimensional approaches in mental health subgroup identification.

Empirical LPA/LCA studies consistently identify 3 ~ 5 mental health profiles. Three-profile solutions are common: Jiang et al. [7] identified *flourishing* (low psychopathology, high well-being), *vulnerable* (low psychopathology, low well-being), and *troubled* (high psychopathology, low well-being) among Chinese university students. Zhou et al. [16] replicated this tripartite structure in Chinese early adolescents, with proportions 48.8%, 39.8%, and 11.4% respectively. Notably, neither of these studies identified a *symptomatic but content* group. Clark and Malecki [24] reported three profiles in U.S. adolescents, including *symptomatic but content* but no *vulnerable* group. Four or five-profile solutions also exist. Kim et al. [20] identified four subtypes (*flourishing*, *moderate flourishing*, *moderate languishing*, *languishing*) in Korean primary students, with gender-specific proportions (14%, 29%, 40%, and 17% among males, and 17%, 38%, 38%, and 8% among females, respectively). Moore et al. [18] found four profiles in U.S. middle schoolers: *complete mental health*, *moderately mentally healthy*, *symptomatic but content*, and *troubled*, with proportions varying by grade. Differently, Gregory et al. [21] reported five profiles, adding a *good mental health* category, namely: *complete mental health* (23%), *good mental health* (33%), *moderate mental health* (27%), *symptomatic but content* (9%), and *troubled* (8%).

Despite advancements, inconsistent profile numbers persist, driven by sample diversity and DFM indicator variability. Specifically, differing operationalizations of well-being (e.g., self-esteem, life satisfaction) and psychopathology (e.g., depression, anxiety) cause divergent profile labels. This highlights the need for systematic LPA exploration of DFM-based mental health profiles across diverse samples or indicators.

### Gender differences in adolescent mental health

Consistent gender differences in adolescent mental health are well-documented: females report more internalizing symptoms (depression, anxiety), with meta-analyses confirming a significant, early-adolescence onset gender gap [25] and faster depressive symptom increases during teenage transitions [3]. Males exhibit higher externalizing behaviors (conduct problems, hyperactivity) [26], with these differences persisting after adjusting for socioeconomic and family factors [1].

LPA refines these differences by identifying gender-patterned subgroups. Most studies identify three to five profiles (low symptoms, internalizing, externalizing, comorbid), with consistent gender disparities characterized by females being overrepresented in internalizing subgroups and males in externalizing subgroups [27]. However, direct research on cross-gender profile structural equivalence remains limited, with mixed findings on measurement invariance. Gregory et al. [21] found identical profile numbers across genders but differing symptom severity thresholds and profile sizes. Polujanski et al. [11] identified gender-specific profiles (e.g., *high internalizing with physical symptoms* in females), suggesting structural comparability but symptomatic/prevalence differences. These findings show subgroup distribution gender differences are consistent, but cross-gender profile structural consistency remains unconfirmed.

DFM-specific cross-gender research is scarce and inconsistent. Petersen et al. [17] found structurally analogous four-class solutions but marked proportional differences (females in emotional symptoms/high well-being; males in conduct problems/high well-being). In contrast, Zhou et al. [16] failed to establish full DFM measurement invariance in Chinese adolescents. They examined Chinese male and female adolescents, found that although the same number of profiles emerged in both groups, symptom and well-being indicators exhibited differential functioning across genders. Compounding these inconsistencies, a systematic review attributed these inconsistencies to heterogeneous designs and insufficient measurement invariance testing [8].

In summary, adolescent mental health gender differences exist, but cross-gender profile structural equivalence remains contested due to methodological flaws. DFM-LPA studies should expand to diverse populations and prioritize measurement invariance validation to distinguish true differences from measurement artifacts.

### Current study

Based on the above review, the present study uses LPA (and multiple-group LPA) within the DFM framework, integrating positive psychological and psychopathological indicators, to address two objectives: (1) identify mental health latent profiles among Chinese adolescents; (2) examine gender differences in these profiles.

## Methods

### Participants

We administered questionnaires to 1,800 adolescents at public schools in Guizhou Province, China. 1,763 questionnaires were returned, resulting in a response rate of 97.94%. After excluding 81 invalid responses (e.g., patterned answering or incomplete data), the final analytic sample consisted of 1,682 valid cases, including 930 males, 724 females, and 28 with missing gender information. The sample had a mean age of 14.37 years (SD = 1.23), ranging from 12 to 18 years. Data collection was conducted in March 2025. Prior to participation, written informed consent was obtained from all students and their legal guardians, with participants informed of their right to withdraw at any time without consequences. Participation was voluntary, anonymous, and unpaid. During the survey, participants completed two standardized questionnaires and a demographic survey.

### Measures

#### Demographic survey

The demographic questionnaire comprised key variables including gender, age, grade, and family location.

#### Center for epidemiological studies depression scale

The *Center for Epidemiological Studies Depression Scale* (*CES-D*), developed by Radloff [28], consists of 20 items, which were originally categorized into four symptom groups: depressed affect (DA), somatic complaints (SC), interpersonal problems (IP), and positive affect (PA). The respondents reported how frequently they experienced each of the symptoms during the past week using a 4-point scale with 1 (rarely or less than 1 day) to 4 (most or all of the time or 5 ~ 7 days). The total score is derived by summing all 20 items, with a possible range of 20 to 80 points; higher scores correspond to more severe depressive symptoms.

For Chinese adolescents, the *CES-D* can be parsed into three symptom dimensions: somatic complaints (SC) comprising 10 items, depressed affect (DA) comprising 6 items, and positive affect (PA) comprising 4 reverse-coded items [29]. Accordingly, the three-factor structure of the *CES-D* was adopted for the present study. The Cronbach’s α coefficients for the total *CES-D* scale and its three dimensions (SC, DA, PA) were 0.863, 0.844, 0.748, and 0.680, respectively and the CFA of three-factor model shows that χ^2^/*df* = 4.700, *SRMR* = 0.035, *CFI* = 0.929, *TLI* = 0.919, *RMSEA* = 0.047 in the present study, indicating good reliability and validity.

#### Satisfaction with life scale

The *Satisfaction With Life Scale* (*SWLS*), developed by Pavot and Diener, comprises five items that participants rated on a 7-point Likert-type scale, ranging from 1 (strongly disagree) to 7 (strongly agree). Total score range from 5 to 35, with higher scores reflecting greater life satisfaction [30, 31]. The present study utilizes the score of the *SWLS* as well-being indicators in the DFM of mental health. The Cronbach’s α coefficient for the *SWLS* was 0.856, and the CFA shows that χ^2^/*df* = 4.789, *SRMR* = 0.010, *CFI* = 0.996, *TLI* = 0.991, *RMSEA* = 0.048 in the present study, demonstrating good internal consistency reliability and validity.

### Statistical analysis

Descriptive statistics for the *CES-D* components and *SWLS* scores were computed using SPSS 26.0, and missing values were handled via listwise deletion in the same software. Additionally, independent samples *t*-tests were conducted to examine gender differences in the continuous variables, while χ^2^ tests were used for demographic categorical variables.

Given the presence of missing values in the gender variable, the current study examined the differences in SC, DA, PA and SWLS scores between participants with complete gender information and those with missing gender information. Independent-samples *t*-tests revealed no significant differences in the four variables between the two groups (all *p*s > 0.05). Consequently, only the data from participants with complete gender information were included in the subsequent latent profile analysis.

The Little’s MCAR test yielded a significant chi-square statistic (χ² = 488.566, *p* < 0.001), indicating that the missing data were not missing completely at random. Based on the z-standardized scores of the three *CES-D* subscales and the *SWLS*, LPA and multiple-group LPA were conducted in M*plus* 8.3 using full information maximum likelihood (FIML) estimation with robust standard errors to empirically identify latent mental health profiles among adolescents, with a robustness check further performed via missing value imputation. Standardization was applied because the three *CES-D* subscales and the *SWLS* have disparate score ranges and unequal metric scales, and the interpretation of latent profiles would lack clarity if raw scores were used for analysis, which ensured the latent profiles were interpretable and readily comparable.

In line with prior methodological recommendations [32–35], the optimal number of latent profiles was identified based on a comprehensive set of fit indices and criteria, including the Akaike Information Criterion (AIC), Bayesian Information Criterion (BIC), sample-adjusted BIC (aBIC), Lo-Mendell-Rubin likelihood ratio test (LMR), bootstrapped likelihood ratio test (BLRT), entropy, and the smallest class size threshold. To verify the stability of LPA results derived from FIML estimation, the present study conducted a robustness check using multiple imputation (MI). Consistent with simulation-based evidence indicating that 20 imputations are appropriate for the majority of empirical scenarios [36], the average values of AIC, BIC, aBIC, and entropy across 20 imputed datasets were computed to implement this robustness assessment.

Gender differences in the latent profiles of mental health were examined through a series of nested model comparisons encompassing an unrestricted model, a partially restricted model, and a fully restricted model, with statistical inferences drawn via likelihood ratio tests (LRT) [37] to evaluate the goodness-of-fit differences between successive nested models.

## Results

### Characteristics of the participants

Among the 1,682 cases, missing data were reported for 28 participants (1.66% of the total sample) on gender, 6 participants (0.36%) on grade, and 216 participants (12.84%) on family location. Table 1 presents the demographic and psychological characteristics of the sample, including the sample size and percentage of the total, as well as the mean scores and standard deviations for all variables. Additionally, it reports the results of gender-based difference tests for each variable.

**Table 1:** Characteristics of the participants

Variables	*N*(%)/Mean(SD)		χ^2^/t	*p*
	Male	Female		
Age	14.44(1.21)	14.25(1.24)	-3.07	< 0.01
Grade				
Grade 1	252(15.29)	276(16.75)	25.91	< 0.001
Grade 2	250(15.17)	186(11.29)		
Grade 3	426(25.85)	258(15.66)		
Family location				
Countryside	478(33.06)	326(22.54)	7.03	< 0.01
Urban	337(23.31)	305(21.09)		
Somatic complaints	6.04(4.98)	8.12(6.51)	6.95	< 0.001
Depressed affect	3.65(3.14)	4.20(3.45)	3.28	< 0.01
Positive affect	3.86(2.49)	4.27(2.54)	3.25	< 0.01
Satisfaction with life	23.00(7.35)	20.20(7.24)	-7.71	< 0.001

### Identification of mental health profiles

#### Mental health profiles for overall samples

The results of LPA are displayed in Table 2. Values of the AIC, BIC, aBIC, entropy, and smallest class size collectively indicate that the three-profile solution is the most optimal for the current dataset. The model demonstrated good classification quality, with average posterior probabilities for each profile being 0.945, 0.880, and 0.906, respectively. To test for local optimal solutions, we employed 5000 random start values and 500 final-stage optimizations. The results showed that the best log-likelihood value was replicated, indicating that this solution was highly likely to be the globally optimal solution with high stability.

**Table 2:** Fit statistics, classification indices, and class sizes for each model

Class	AIC	BIC	aBIC	Entropy	LMR(*p*)	BLRT(*p*)	Class Proportions
2	16651.21	16721.55	16680.25	0.822	< 0.001	< 0.001	0.742/0.258
3	**16245.57**	**16342.97**	**16285.79**	**0.828**	**< 0.001**	**< 0.001**	**0.625/0.305/0.070**
4	16105.03	16229.49	16156.42	0.817	< 0.05	< 0.001	0.540/0.316/0.038/0.106
5	15956.52	16108.02	16019.07	0.751	< 0.05	< 0.001	0.148/0.487/0.174/0.043/0.148

The bolded row in the table represents the optimal latent class

**Fig. 1:**
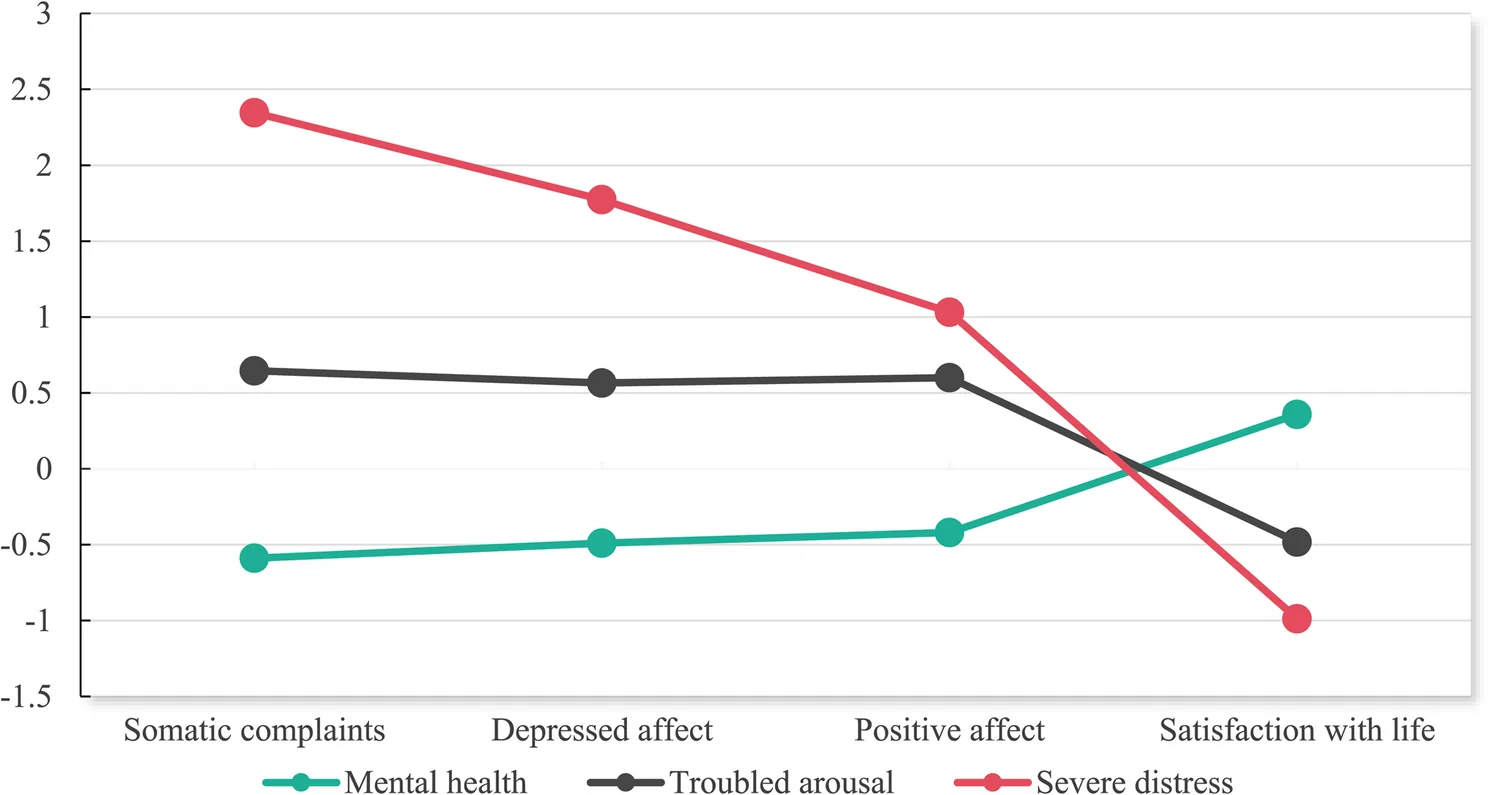
Latent profiles of mental health based on dual-factor model

Figure 1 presents estimated mean plots for the three-profile solution in the current dataset. Based on the distinct patterns of standardized scores across the symptom dimensions and life satisfaction, the three latent profiles identified in this study were labeled as follows:

##### Mental health profile

The largest profile, comprising 1033 participants (62.5% of the total sample), was characterized by significantly negative standardized scores across all distress-related dimensions: somatic complaints (-0.589), depressed affect (-0.489), and positive affect (-0.420). In contrast, this group exhibited a positive score on satisfaction with life (0.359), reflecting low levels of psychological distress and high life satisfaction, indicative of a well-adapted mental health state.

##### Troubled arousal profile

The second-largest profile, consisting of 505 participants (30.5% of the sample), displayed moderately positive standardized scores on somatic complaints (0.646), depressed affect (0.565), and positive affect (0.601), alongside a mildly negative score on satisfaction with life (-0.482). This pattern suggests a persistent state of subclinical psychological distress and emotional arousal, with mild impairment in life satisfaction.

##### Severe distress profile

The smallest profile, including 116 participants (7.0% of the sample), was marked by extremely elevated positive standardized scores on somatic complaints (2.346), depressed affect (1.773), and positive affect (1.032), paired with a strongly negative score on satisfaction with life (-0.988). These individuals exhibited severe psychological distress across all symptom domains and profound dissatisfaction with life, representing a clinically significant level of mental impairment.

To assess the robustness of the LPA results across the full sample, this study employed MI with 20 imputed datasets, comparing the findings to those derived from FIML estimation. For each imputed dataset, model fit indices were computed including AIC, BIC, aBIC, and entropy, along with the proportion of participants assigned to each profile, then averaged these values across all 20 imputations (Table 3). As shown in Table 3, the results from the MI approach were highly consistent with those from FIML estimation, with substantial agreement in the optimal number of profiles, entropy values, and profile class proportions. This consistency confirms the robustness of the LPA results to missing data, supporting the reliability of the profile structure identified.

**Table 3:** Fit statistics, classification indices, and class sizes for the robust test of each model

Class	Mean AIC	Mean BIC	Mean aBIC	Mean Entropy	Class Proportions
2	17229.12	17299.47	17258.17	0.830	0.740/0.260
3	**16813.91**	**16911.30**	**16854.12**	**0.837**	**0.619/0.310/0.071**
4	16679.38	16803.83	16730.76	0.822	0.527/0.317/0.037/0.119
5	16517.05	16668.56	16579.60	0.759	0.172/0.162/0.476/0.148/0.042

The bolded row in the table represents the optimal latent class

#### Mental health profiles for male and female samples respectively

Two separate LPA models with 2- to 5-class were conducted on male and female samples to determine whether an identical number of mental health profiles derived from each sample.

**Fig. 2:**
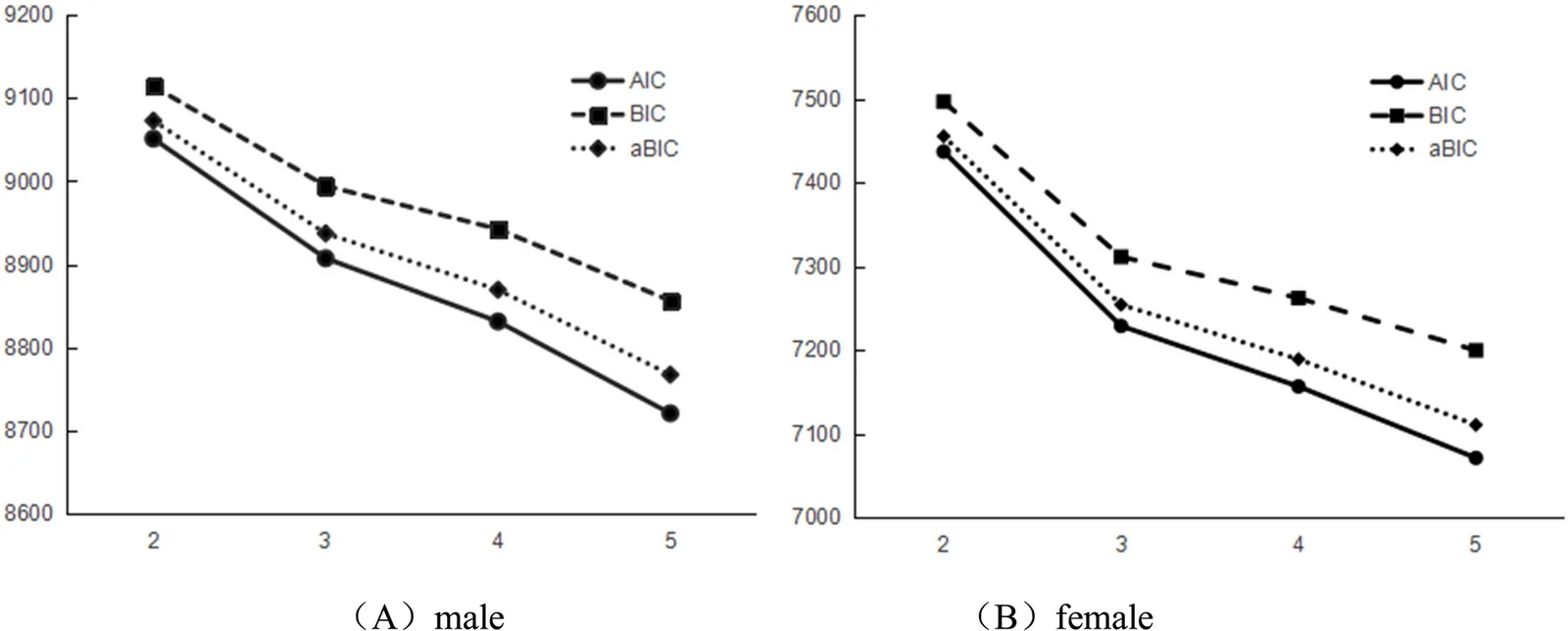
Information criteria change patterns of different profile solutions across gender

As presented in Table 4; Fig. 2, for the male subsample, the 4-class solution was not adopted because it resulted in lower entropy and a non-significant LMR test. Similarly, the 5-class solution was rejected due to its low entropy value and the presence of a very small, less interpretable class, therefore, the 3-class model was identified as the optimal solution based on entropy, AIC, BIC, aBIC and profile proportions with average posterior probabilities for each profile being 0.943, 0.863, and 0.875, respectively.

**Table 4:** Model fit indices of mental health profiles for male and female samples

Gender	Class	AIC	BIC	aBIC	Entropy	LMR(*p*)	BLRT(*p*)	Class Proportions
Male	2	9050.96	9113.82	9072.53	0.815	< 0.001	< 0.001	0.723/0.277
	**3**	**8907.14**	**8994.17**	**8937.01**	**0.820**	**< 0.05**	**< 0.001**	**0.630/0.299/0.071**
	4	8831.09	8942.30	8869.25	0.760	0.062	< 0.001	0.157/0.545/0.180/0.118
	5	8720.73	8856.12	8767.19	0.777	< 0.001	< 0.001	0.074/0.156/0.243/0.485/0.042
Female	**2**	**7436.63**	**7496.23**	**7454.95**	**0.839**	**< 0.001**	**< 0.001**	**0.725/0.275**
	3	7228.57	7311.10	7253.94	0.814	< 0.001	< 0.001	0.564/0.333/0.103
	4	7156.42	7261.87	7188.84	0.812	< 0.05	< 0.001	0.510/0.130/0.304/0.056
	5	7071.04	7199.42	7110.51	0.775	0.199	< 0.001	0.463/0.157/0.193/0.130/0.057

The bolded rows in the table represent the optimal latent classes for male and female samples, respectively

**Fig. 3:**
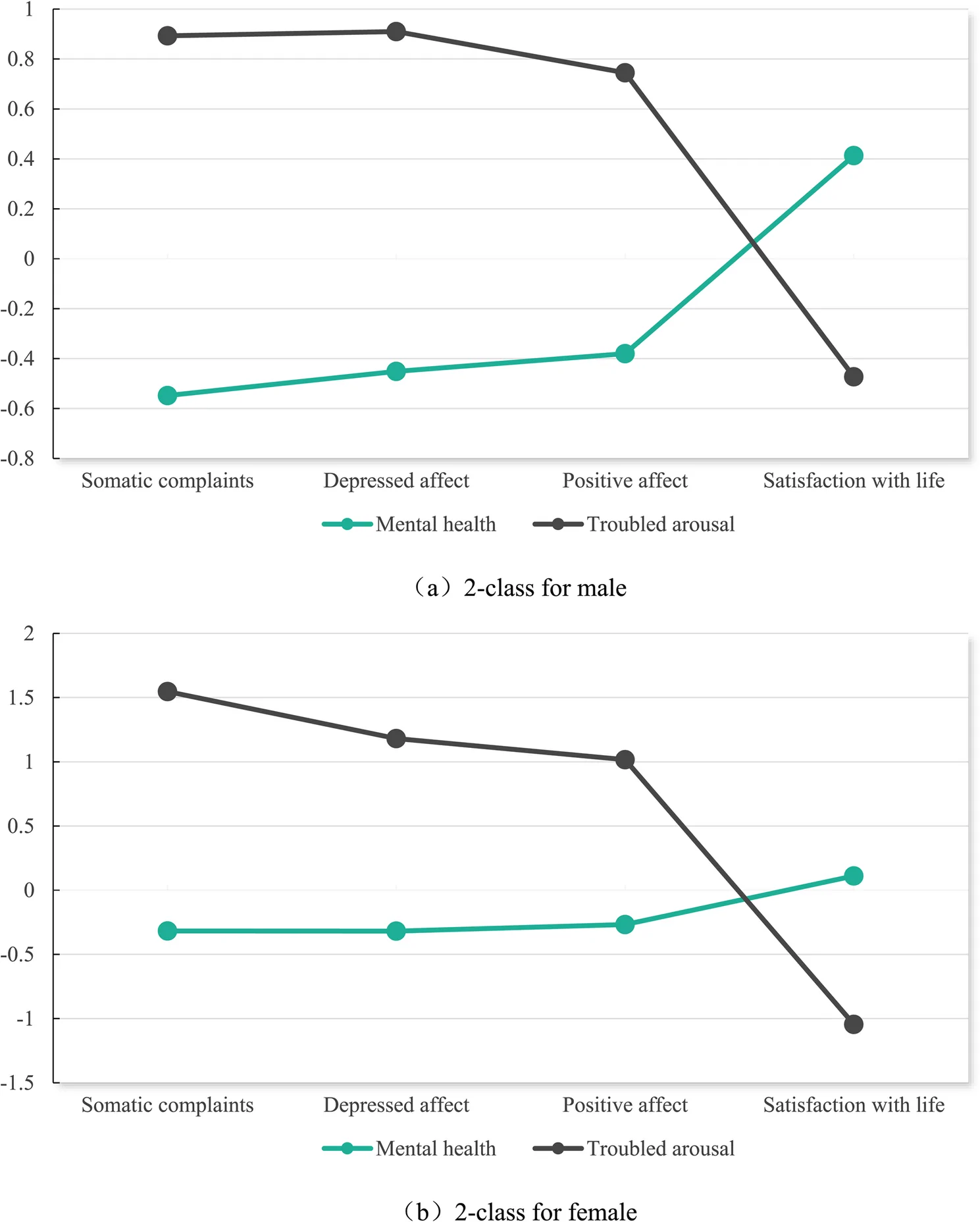
Two- profile solutions across gender

**Fig. 4:**
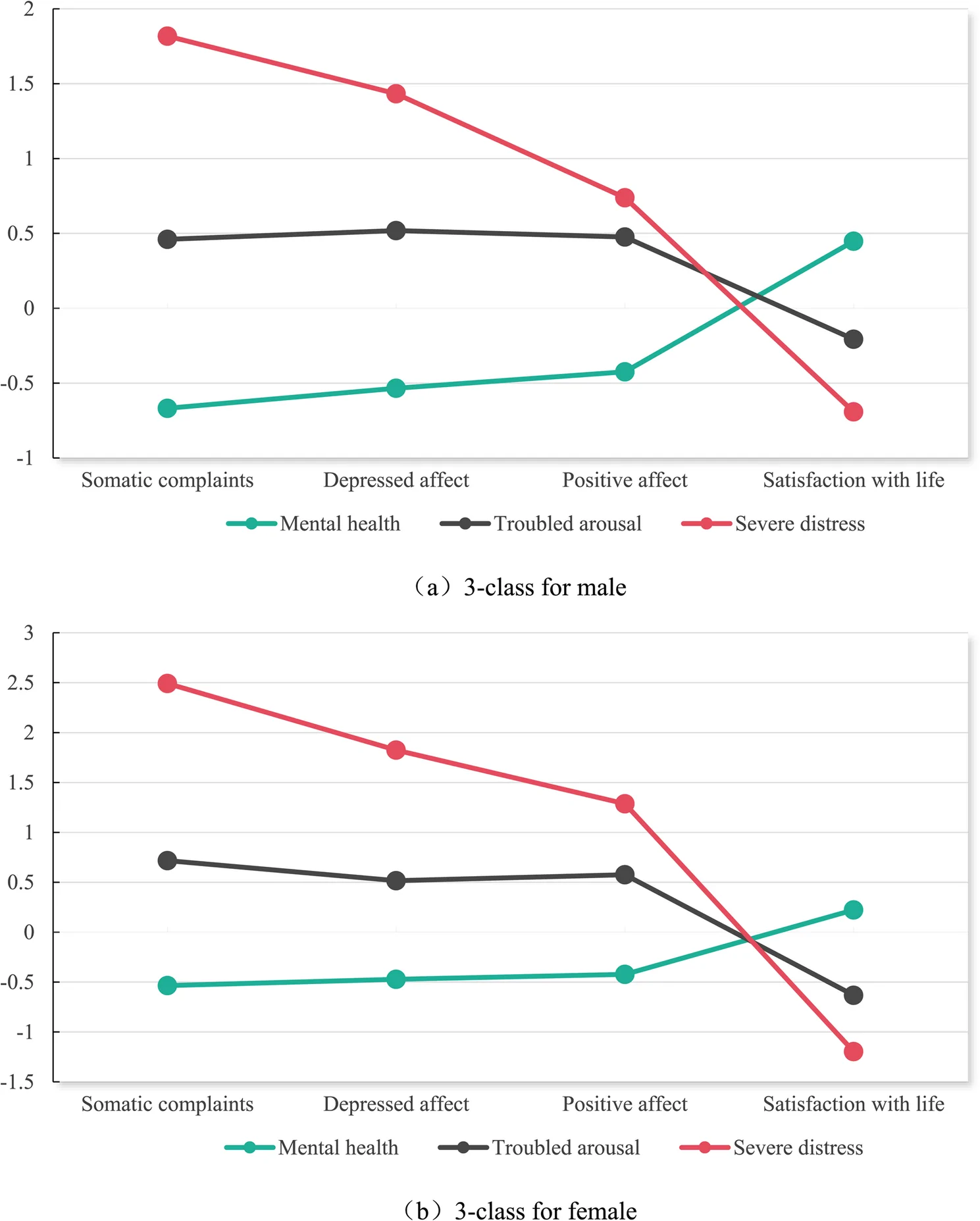
Three- profile solutions across gender

However, for the female subsample, although the 3-class and 4-class models yielded notably lower AIC, BIC, and aBIC values, significant LMR test results, and no extremely small classes, suggesting a more complex underlying structure, the additional classes in these solutions still showed insufficient distinctness and stability compared with the more parsimonious 2-class model. Given that a higher entropy value enables more accurate and stable classification of profiles, the 2-class solution was therefore selected as the optimal one for females. Therefore, the 2-class model was optimal solution for the female sample with average posterior probabilities for each profile being 0.969, and 0.923, respectively. Figures 3 and 4 show 2- and 3- profile solutions across gender respectively.

Furthermore, the results of the multiple imputation analysis for handling missing data, as presented in Table 5, are highly consistent with the current findings, supporting the stability of the identified profile solutions.

**Table 5:** Model fit indices of mental health profiles for males and females (robust test)

Gender	Class	Mean AIC	Mean BIC	Mean aBIC	Mean Entropy	Class Proportions
Male	2	9405.36	9468.22	9426.94	0.828	0.721/0.279
	**3**	**9251.08**	**9338.11**	**9280.94**	**0.831**	**0.626/0.299/0.075**
	4	9167.31	9278.52	9205.48	0.771	0.180/0.121/0.544/0.155
	5	9069.72	9205.11	9116.18	0.782	0.485/0.147/0.083/0.239/0.046
Female	**2**	**7665.53**	**7725.13**	**7683.85**	**0.842**	**0.724/0.276**
	3	7450.92	7533.45	7476.29	0.823	0.336/0.560/0.104
	4	7379.67	7485.12	7412.09	0.780	0.187/0.525/0.189/0.099
	5	7292.98	7421.36	7332.45	0.782	0.445/0.204/0.173/0.122/0.056

The bolded rows in the table represent the optimal latent classes for male and female samples, respectively

Single-group LPA revealed a three-profile solution for the overall sample, whereas separate LPAs for males and females yielded discrepant profile numbers. Though this cross-gender heterogeneity in profile count would conventionally conclude the multiple-group LPA process, additional comparative analyses were performed to explore gender differences in mental health latent profiles under both the two-profile and three-profile structural frameworks for more thorough gender-specific inferences.

In the multiple-group LPA, both male and female samples were represented by a 2-profile solution, which were consistently named the mental health profile and troubled arousal profile across genders (Fig. 3). For males, the mental health profile included 672 participants (72.3% of the male sample), while the troubled arousal profile comprised 258 participants (27.7%). For females, the mental health profile accounted for 525 participants (72.5% of the female sample), and the troubled arousal profile included 199 participants (27.5%). Notably, the proportional distribution of the two profiles was highly similar between genders, with the mental health profile being the dominant subgroup in both groups, but significant gender differences perhaps emerged in the mean scores of symptom dimensions within the troubled arousal profile. Females exhibited substantially higher standardized scores on somatic complaints (1.547 vs. 0.893), depressed affect (1.181 vs. 0.91), positive affect (1.016 vs. 0.745), and more severe dissatisfaction with life (-1.045 vs. -0.473) compared to males.

Additionally, in the multiple-group LPA, both male and female adolescents were characterized by a 3-profile solution, consistent with the total sample, which were consistently labeled the mental health profile, troubled arousal profile, and severe distress profile across genders. For males, the mental health profile included 586 participants (63.0% of the male sample), the troubled arousal profile comprised 278 participants (29.9%), and the severe distress profile included 66 participants (7.1%). For females, the mental health profile accounted for 408 participants (56.4% of the female sample), the troubled arousal profile included 241 participants (33.3%), and the severe distress profile comprised 75 participants (10.3%). While the mental health profile was the dominant subgroup in both genders, notable gender differences perhaps emerged in both profile proportions and mean scores: females exhibited a larger proportion of individuals in the distressed profiles (troubled arousal and severe distress) and showed substantially higher standardized scores on all symptom dimensions within the severe distress profile, including somatic complaints (2.492 vs. 1.818), depressed affect (1.825 vs. 1.433), positive affect (1.287 vs. 0.738), and more severe dissatisfaction with life (-1.196 vs. -0.692).

To assess the robustness of the LPA results across the male and female samples, this study also employed MI with 20 imputed datasets. When conducted separately by gender, multiple imputation also yielded a 3-profile solution for males and a 2-profile solution for females (Table 5), which were consistent with the FIML results.

#### Model comparison of multiple-group LPA for mental health

Five multiple-group LPAs were conducted to examine whether the 2- and 3-profile solutions identified separately for male and female samples exhibited an identical profile structure. Model fit indices are presented in Tables 6 and 7.

**Table 6:** Model fit indices of multiple-group LPA models for the 2-profile solution

Model	Free parameters	LL	AIC	BIC	aBIC	LRT (χ^2^)
Unconstrained	35	−9110.97	18291.94	18481.32	18370.13	-
Mean-constrained	27	−9171.06	18396.11	18542.21	18456.44	120.18^***^
Profile size-constrained	34	−9111.42	18290.84	18474.82	18366.80	0.90
Fully constrained	18	−9196.19	18428.39	18525.78	18468.60	170.44^***^

***p <0.001

**Table 7:** Model fit indices of multiple-group LPA models for the 3-profile solution

Model	Free parameters	LL	AIC	BIC	aBIC	LRT (χ^2^)
Unconstrained	53	−8833.17	17772.34	18059.12	17890.75	-
Mean-constrained	41	−8910.03	17902.06	18123.91	17993.66	153.72^***^
Profile size-constrained	51	−8846.23	17794.45	18070.41	17908.39	26.12^***^
Fully constrained	27	−8940.08	17934.16	18080.25	17994.48	213.82^***^

***p <0.001

For the 2-profile solution, this study performed nested model comparisons using the LRT to assess the impact of cross-group constraints on model fit (Table 6). Compared to the unconstrained model, the mean-constrained model showed a significant deterioration in fit (χ^2^ = 120.18, *p* < 0.001), indicating that constraining means to be equal across profiles significantly worsens model fit and thus is not justified, however, the profile size-constrained model yielded a non-significant chi-square value (χ^2^ = 0.90, *p* = 0.34), suggesting that constraining profile sizes to be equal across groups does not significantly compromise model fit and is a more parsimonious and acceptable specification. The fully constrained model showed a significant deterioration in fit (χ^2^ = 170.44, *p* < 0.001).

For the 3-profile solution, this study also conducted nested model comparisons using the LRT to evaluate the effect of cross-group constraints on model fit (Table 7). When compared to the unconstrained model, the mean-constrained model exhibited a significant deterioration in fit (χ^2^ = 153.72, *p* < 0.001), indicating that constraining means to be equal across profiles significantly impairs model fit and is therefore not warranted and the profile size-constrained model yielded a significant chi-square value (χ^2^ = 26.12, *p* < 0.001), suggesting that constraining profile sizes to be equal across groups significantly compromises model fit and is not a justifiable specification. The fully constrained model also showed a significant deterioration in fit (χ^2^ = 213.82, *p* < 0.001).

## Discussion

This study employed LPA and multiple-group LPA within the DFM framework to identify mental health profiles among Chinese adolescents and examine gender differences in these profiles. The findings revealed three distinct mental health profiles in the total sample, with notable gender variations in profile structure, proportions, and symptom severity.

In the total sample, three latent profiles were identified. The mental health profile (62.5%) was characterized by low psychological distress across somatic complaints, depressed affect, and positive affect, alongside high life satisfaction, indicating a well-adapted mental health state. The troubled arousal profile (30.5%) was marked by moderate subclinical distress and mild impairment in life satisfaction, representing a state of persistent emotional arousal. The severe distress profile (7.0%) exhibited severe psychological distress across all domains and profound life dissatisfaction, reflecting clinically significant mental impairment.

Gender-specific analyses revealed structural differences: males were classified into three profiles consistent with the total sample, while females were optimally categorized into two profiles. Comparative analysis of three-profile solutions across genders further showed that females had a larger proportion of individuals in distressed profiles (43.6% vs. 37.0% in males) and significantly higher symptom severity in the severe distress profile, particularly in somatic complaints, depressed affect, and life dissatisfaction. These results align with the DFM’s emphasis on integrating both psychopathological symptoms and positive well-being to enable an integrated assessment of mental health [4].

The three-profile solution in the total sample is consistent with several prior studies using LPA within the DFM framework. For example, Jiang et al. identified three profiles among Chinese university students [7], and Zhou et al. reported a tripartite structure in Chinese early adolescents [16]. However, other studies have found four or five profiles. Kim et al. identified four subtypes, including flourishing, moderate flourishing, moderate languishing, languishing, in Korean primary school students [20], while Gregory et al. reported five profiles in Australian school students [21]. In comparison with the four-category classification of the DFM, two of the three profiles identified in the present study align with the *complete mental health* and *troubled* subtypes of the DFM’s four categories, with the *vulnerable* and *symptomatic but content* subtypes absent. There are also discrepancies in the nomenclature of latent profiles relative to other LPA-based studies. Variations in profile numbers and nomenclature across studies may stem from differences in sample characteristics, measurement tools, e.g., inclusion of multiple psychopathological or well-being indicators, and analytical approaches. This may also be attributable to the specific selection of assessment dimensions in the current research, the age and developmental characteristics of the study sample, and the varying threshold criteria for profile classification in LPA analyses. The current study’s focus on only depressive symptoms and life satisfaction may have contributed to fewer profiles compared to studies incorporating additional indicators such as anxiety or self-esteem.

The gender-specific profile differences, three profiles for males and two for females, add to the growing literature on gender heterogeneity in mental health profiles. While some studies have found identical profile structures across genders [17], others have reported gender-specific profiles [11], suggesting that gender differences may manifest in both symptom severity and underlying profile structure. The proportion of adolescents in the mental health profile (62.5%) is higher than reported in some prior studies. Jiang et al. found 45.6% of Chinese university students in the complete mental health profile [7], and Zhou et al. reported 48.8% of Chinese early adolescents in the flourishing profile [16]. This discrepancy may reflect age-related trends, as older adolescents or young adults may face more mental health challenges compared to middle school students [3]. Gender differences in profile proportions and symptom severity align with extensive prior research. Females were overrepresented in distressed profiles and exhibited higher mean scores on all depressive symptom dimensions and lower life satisfaction than males. These findings support meta-analyses showing females report more internalizing symptoms during adolescence [25] and cross-national studies documenting a gender gap in adolescent mental health. The greater symptom severity in females within the severe distress profile underscores the need for gender-specific interventions.

The identification of distinct mental health profiles may offer potentially useful implications for understanding adolescent mental health in China. First, the identified profiles may help categorize adolescents into low-, moderate-, and high-risk groups, which could inform future targeted prevention and intervention efforts. Second, females, particularly those in the severe distress profile, appeared to exhibit more severe psychological distress, suggesting that gender-tailored support may warrant further attention in future clinical and school-based practices. Third, gender-sensitive screening strategies in school settings may be worth exploring in future mental health programs. Finally, the DFM-based LPA approach may serve as a person-centered complement to traditional symptom-focused screenings, which could help identify at-risk adolescents who might otherwise be overlooked by conventional approaches.

## Limitations and future directions

This study is subject to several notable limitations. First, the cross-sectional design precludes causal inferences about the relationships between latent mental health profiles and their associated influencing factors, as it cannot capture the temporal dynamics of these associations. Second, the sample was recruited solely from Guizhou Province, which restricts the generalizability of the findings and limits the ability to extend conclusions to adolescents from other geographic, socioeconomic, or ethnic backgrounds across China. Finally, the measurement scope was narrow, being limited to depressive symptoms and life satisfaction; the exclusion of additional multidimensional mental health indicators, such as anxiety, externalizing behaviors, and social well-being, prevents a fully comprehensive and nuanced characterization of adolescent mental health profiles within the DFM framework. In addition, regarding missing data handling, although FIML and multiple imputation were employed under the Missing at Random (MAR) assumption, Little’s MCAR test was significant, indicating that data were not missing completely at random. If some missingness were in fact Missing Not At Random (MNAR), this could potentially introduce bias into the parameter estimates.

Accordingly, several targeted directions for future research are proposed. First, longitudinal research designs are needed to track the stability of identified latent profiles over time and identify key predictors of profile transitions. Second, future studies should expand the sampling scope to include diverse adolescent groups across China, encompassing different geographic regions, socioeconomic strata, and ethnic groups. Finally, the measurement system should be enriched by integrating a broader set of positive psychological indicators (e.g., psychological and social well-being) and comprehensive psychopathological symptoms (e.g., anxiety, externalizing behaviors) to capture the full multidimensionality of mental health and identify more refined latent profiles that may have been overlooked in the present study.

## Conclusion

This study identified three distinct mental health profiles among Chinese adolescents, with significant gender differences in profile structure, proportions, and symptom severity. Females were disproportionately represented in distressed profiles and exhibited greater symptom severity, highlighting the need for gender-sensitive mental health interventions. These findings advance our understanding of adolescent mental health in China and provide a foundation for targeted screening, prevention, and intervention strategies. While limitations exist, the study demonstrates the utility of DFM-based LPA for targeted mental health assessment within a streamlined indicator set and underscores the importance of considering gender in adolescent mental health research.

## Data Availability

The data used to support the findings of this study are available from the corresponding author upon request.
